# Iron Supplementation at 1 mg/kg/Day for Exclusively Breastfed Full-Term Infants: A Meta-Analysis

**DOI:** 10.1155/ijpe/6662278

**Published:** 2025-09-17

**Authors:** Suyu Wang, Yayu Zhang, Dan Song, Hua Mei, Zhiying You, Xiaofang Xu, Yuqi Zhang

**Affiliations:** ^1^First School of Clinical Medicine, Inner Mongolia Medical University, Hohhot, China; ^2^Department of Neonatology, The Affiliated Hospital of Inner Mongolia Medical University, Hohhot, China

**Keywords:** breastfeeding, infants, iron supplementation, meta-analysis

## Abstract

**Aim:** The aim for the study is to evaluate the effects of 1 mg/kg/day iron supplementation on hematologic and growth parameters in exclusively breastfed full-term infants.

**Methods:** We conducted a meta-analysis of seven RCTs (*n* = 1114) comparing iron supplementation with placebo/no intervention. Primary outcomes included hemoglobin (Hb), serum ferritin (SF), mean corpuscular volume (MCV), height, and weight. Data were analyzed using RevMan 5.4.

**Results:** Iron supplementation from 4 to 6 months significantly increased Hb (MD = 6.29 g/L, *p* = 0.01), but effects diminished with longer duration (MD = 2.00 g/L, *p* = 0.08). For SF, no significant difference was found at 6 months, but supplementation significantly increased SF at 12 months (MD = 5.95 *μ*g/L, *p* = 0.04). No significant differences were found in MCV, height, or weight. Subgroup variations were noted by formulation and region.

**Conclusion:** While short-term iron supplementation improves iron status, the lack of sustained benefits suggests individualized approaches may be preferable to universal supplementation in well-nourished populations.

## 1. Introduction

Current guidelines predominantly advise initiating iron supplementation at 1 mg/kg/day from 4 months of age in exclusively breastfed full-term infants. However, debate persists concerning ideal dosage and initiation timing. Iron deficiency anemia (IDA) represents the most prevalent form of nutritional anemia globally, especially affecting infancy. While breast milk is the optimal infant food with high iron bioavailability, its iron concentration remains low (3–4.5 mg/L). The physiological iron requirement during the first 6 months of life is approximately 0.3 mg/day, while breast milk alone cannot sufficiently provide [1]. Term infants typically possess endogenous iron stores (approximately 75 mg/kg) at birth. However, these reserves generally deplete by 4–6 months of age, and the bioavailability of iron in breast milk is only 10%–20% [2]. Consequently, exclusively breastfed infants without iron supplementation face elevated risks of developing iron deficiency (ID) and IDA. Such deficiencies may lead to irreversible impairments in neurocognitive development, immune function, and motor abilities [3]. The World Health Organization (WHO) advises daily elemental iron supplementation (1–2 mg/kg) for low-risk term infants between 6 and 23 months of age [4]. In contrast, the American Academy of Pediatrics (AAP) recommends 1 mg/kg/day for exclusively breastfed infants starting at 4 months, continuing until iron-fortified complementary foods are introduced [5]. The European Society of Pediatric Gastroenterology, Hepatology, and Nutrition (ESPGHAN) does not consider routine supplementation to be necessary for full-term, normal-weight breastfed infants [6]. Meanwhile, China issued an expert consensus in 2023 recommending that full-term breastfed and mixed-feeding infants be given iron supplements (1 mg/kg/day, maximum 15 mg/day) from 4 months of age for term breastfed and mixed-fed infants, until dietary iron intake becomes sufficient [7]. However, current guidelines remain divided regarding the efficacy of 1 mg/kg/day iron supplementation in preventing ID and IDA in exclusively breastfed term infants. For this reason, the present study systematically evaluates the preventive effect of 1 mg/kg/day iron supplementation on iron status in exclusively breastfed full-term infants through meta-analysis, aiming to establish an evidence-based basis for clinical practice.

## 2. Materials and Methods

### 2.1. Study Protocol and Search Strategy

This systematic review adhered to the Cochrane Handbook for Systematic Reviews and Meta-Analysis protocol (PRISMA-P) guidelines [8] and was registered in PROSPERO (CRD420251059699).

A comprehensive literature search was conducted across multiple databases including PubMed, Embase, Web of Science, China Knowledge Network (CNKI), and Wanfang Data Knowledge Service Platform (Wanfang Data). The search included all publications available before February 15, 2025. Combining the characteristics of each database, the literature was searched using a combination of subject terms and free words. Key search terms comprised Breast Feeding, Infants, Newborn, Newborn Infant, Iron.

### 2.2. Literature Inclusion and Exclusion Criteria

Inclusion criteria followed the PICOS framework (P: population; I: intervention; C: comparison; O: outcome; S: study design). Population: full-term exclusively breastfed infants (gestational age [GA] ≥ 37 weeks); intervention: elemental iron supplementation (1 mg/kg/day); comparison: placebo or no iron supplementation; outcome: hemoglobin (Hb), mean corpuscular volume (MCV), serum ferritin (SF), and growth measures (height, weight); study design: randomized controlled trials (RCTs).

Exclusion criteria are as follows: (1) duplicate or overlapping datasets; (2) non-English and non-Chinese publications; (3) review articles, conference abstracts, and commentaries; (4) studies with flawed methodology or low quality; and (5) incomplete or inaccessible primary data.

### 2.3. Data Extraction

Two researchers (Suyu Wang and Yayu Zhang) independently performed literature screening and data extraction. Cross-validation was performed to ensure the accuracy of the results. Disagreements were resolved through discussion or third-party consultation. The screening process involved (1) initial title review to exclude irrelevant literature, (2) abstract assessment for preliminary eligibility, and (3) full-text evaluation for final inclusion. Extracted information included first author, publication date, study location, iron supplementation dose, intervention duration, and sample sizes (intervention and control groups.) In addition, the mean and standard deviation (SD) were extracted from each of the included studies. Any discrepancies in data extraction were resolved by consensus or by consulting a third reviewer (D.S. or H.M.).

### 2.4. Quality Assessment

Two independent reviewers (Suyu Wang and Yayu Zhang) evaluated study quality using the Cochrane risk of bias (RoB) tool (Review Manager Version 5.4). Each study received ratings of “yes” (low risk), “no” (high risk), or “unclear” (insufficient information) across seven domains. These judgments determined the bias of the literature and the reliability of the outcome.

### 2.5. Statistical Analysis

Meta-analysis employed Review Manager 5.4 to systematically evaluate the impact of 1 mg/kg/day iron supplementation on iron status and development outcomes in exclusively breastfed full-term infants. Continuous variables were analyzed using mean differences (MDs) ± SDs, presented with a 95% confidence interval (CI). Study heterogeneity was assessed through the *I*^2^ test, which indicates between-study heterogeneity when *I*^2^ > 50% and *p* ≤ 0.1, a random-effects model was used, and subgroup or sensitivity analyses were performed to explore potential sources of heterogeneity. Conversely, fixed-effects models were applied when these thresholds were not reached. Differences between the two groups were statistically significant when *p* ≤ 0.05.

## 3. Results

### 3.1. Literature Screening Process and Basic Characteristics of Included Literature

**Figure 1:**
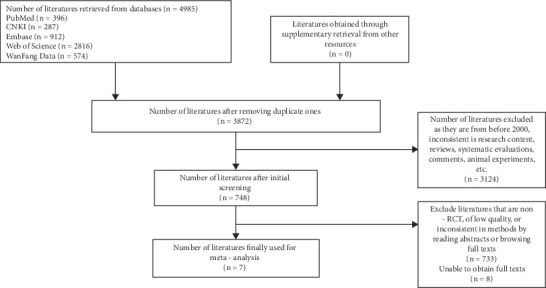
PRISMA flow diagram of study selection.

The initial search yielded 4985 articles. After sequential screening, seven RCTs (9, 10, 11, 12, 13, 14, 15) met the inclusion criteria. Among these studies, Domellöf et al. (2001) reported data separately for two countries, and these were analyzed as distinct dataset. The selection process is detailed in Figure 1. The final analysis included 1114 children. All studies were published post-2000 and employed RCTs. Four studies involved iron supplementation for 4–6 months, with outcome measurements taken at 6 months of age. Three studies provided approximately 6 months of iron supplementation but assessed outcomes at 12 months.  Table 1 summarizes the baseline characteristics of included studies.

### 3.2. Evaluation of the Quality of the Literature

**Figure 2:**
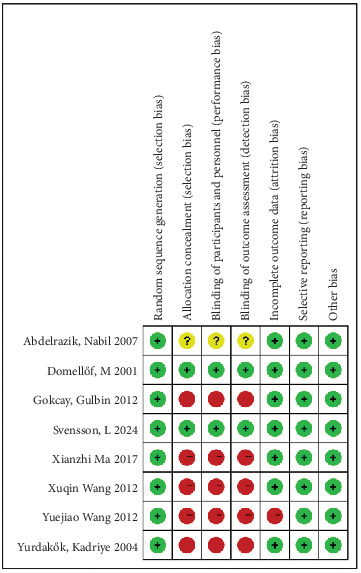
Evaluation of literature quality.

Study quality assessment was performed with the Cochrane Evaluation Handbook. According to the RoB evaluation chart, the seven included studies were evaluated for risk in several aspects as follows: (1) Random sequence generation (selection bias): all trials employed randomized controlled designs and received “low risk.” (2) Assignment concealment (selection bias): two studies [9, 10] were rated as “low risk.” One study [11] lacked a description of concealment methods, resulting in “low risk.” The remaining four studies [12–15] exhibited “high risk.” (3) Blinding of participants and staff (performance bias): two studies were rated as “low risk,” four as “high risk,” and one study failed to report blinding status. (4) Blinding of outcome assessment (detection bias): two studies explicitly implemented blinded outcome assessment and were rated as “low risk.” One study lacked reporting on blinding and was deemed “unclear risk.” The remaining four studies were rated as “high risk.” (5) Incomplete outcome data (missing visit bias): one trial exhibited substantial missing data. The other six studies maintained complete datasets without significant attrition, resulting in “incomplete.” (6) Selective reporting (reporting bias): all seven studies demonstrated no evidence of selective reporting and were rated as “low risk.” (7) Other bias: all seven studies did not report other bias problems and were rated as “low risk.” In summary, the majority of studies exhibited high RoB in allocation concealment, blinding application, and data completeness. Overall methodological quality remained high. The included RoB assessment is presented in Figure 2.

### 3.3. Meta-Analysis Results

#### 3.3.1. Hb

**Figure 3:**
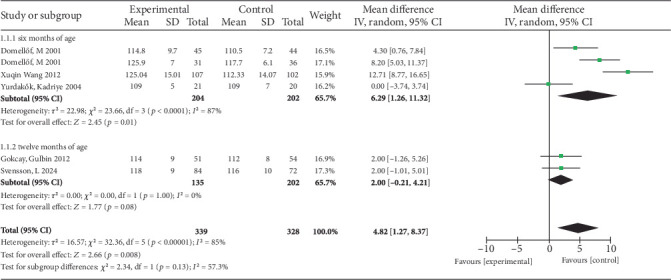
Forest plot for Hb.

Meta-analysis of six studies [9–12, 15] evaluating 1 mg/kg/day iron supplementation in exclusively breastfed full-term infants revealed significant Hb improvement (MD = 4.82 g/L, 95% CI 1.27–8.37, *p* = 0.008) compared to placebo/no supplementation, with substantial heterogeneity (*p* < 0.05, *I*^2^ = 85%). Random-effects modeling was applied. Subgroup analysis demonstrated: From 4 to 6 months of age supplementation (four studies [9, 12, 15]) showed greater Hb increase (MD = 6.29 g/L, 95% CI 1.26–11.32, *p* = 0.01) with high heterogeneity (*p* < 0.05, *I*^2^ = 87%). From 4 to 12 months of age supplementation (two studies [10, 11]) showed a nonsignificant effect (MD = 2.00 g/L, 95% CI −0.21 to 4.21, *p* = 0.08) with no heterogeneity (*p* = 1.00, *I*^2^ = 0%). Fixed-effects modeling was used, and the forest plot is presented in Figure 3.

Subgroup analyses were performed for the intervention period from 4 to 6 months of age group (Table 2). Results were stratified as follows: (1) grouped by iron compound type: ferrous sulfate group (MD = 4.25 g/L, 95% CI −0.42 to 8.92, *p* = 0.07) and iron dextran group (MD = 12.71 g/L, 95% CI 8.77–16.65, *p* < 0.05); (2) grouped by different ethnicities: Asian African (MD = 6.34 g/L, 95% CI −6.12 to 18.79, *p* = 0.32) and European American (MD = 6.33 g/L, 95% CI 2.52–10.15, *p* = 0.001); (3) grouped by sample size: large sample (MD = 8.33 g/L, 95% CI 3.84–12.83, *p* = 0.0003) and small sample (MD = 0 g/L, 95% CI −3.74 to 3.74, *p* = 1); (4) grouped by ID severity: iron insufficiency regions (MD = 5.64 g/L, 95% CI −1.45 to 12.73, *p* = 0.12) and iron sufficiency regions (MD = 8.2 g/L, 95% CI 5.03–11.37, *p* < 0.001).

#### 3.3.2. SF

**Figure 4:**
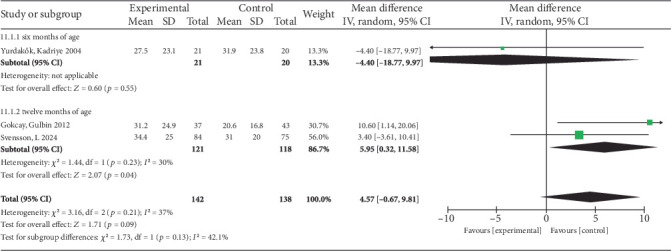
Forest plot for SF.

Three studies were included in the analysis (*I*^2^ = 37). After the iron supplementation intervention, there was no difference in SF levels between the experimental group and the control group (MD = 4.57 *μ*g/L, 95% CI −0.67 to 9.81). At 6 months, a single study found no significant impact of iron supplementation on SF levels (MD = −4.40 *μ*g/L, 95% CI −18.77 to 9.97, *p* = 0.55). However, at 12 months, the pooled analysis of two studies demonstrated a significant positive effect (MD = 5.95 *μ*g/L, 95% CI 0.32–11.58, *p* = 0.04). Consequently, while the overall analysis, influenced by the nonsignificant 6-month result, showed no statistically significant difference, the 12-month subgroup indicates a clear benefit of supplementation. This suggests iron supplementation significantly increases SF specifically in 12-month-old infants but not in those aged 6 months. The relevant charts are shown in Figure 4.

#### 3.3.3. MCV

**Figure 5:**
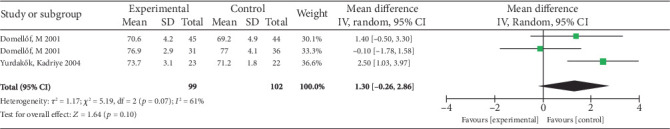
Forest plot for MCV.

Three studies evaluating 1 mg/kg/day iron supplementation from 4 to 6 months of age were included, with MCV outcomes measured at 6 months. Moderate statistical heterogeneity was observed (*p* = 0.07, *I*^2^ = 61%), within acceptable limits. Meta-analysis was performed using a random-effects model, which revealed no statistically significant difference in MCV between iron-supplemented and placebo/nonsupplemented groups (MD = 1.30, 95% CI −0.26 to 2.86, *p* = 0.1), and the forest plot is presented in Figure 5.

#### 3.3.4. Rate of Height Growth (Millimeter/Month)

**Figure 6:**
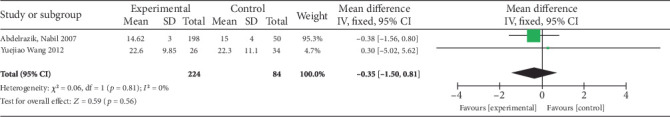
Forest plot for rate of height growth.

Two articles were included in the analysis, demonstrating no heterogeneity (*p* = 0.18, *I*^2^ = 0%). Meta-analysis using a fixed-effects model indicated no statistically significant effect of 1 mg/kg/day iron supplementation compared to placebo/no supplementation on infant monthly height growth (MD = −0.35, 95% CI −1.50 to 0.81, *p* = 0.56), and the forest plot is shown in Figure 6.

#### 3.3.5. Rate of Weight Gain (Kilogram/Month)

**Figure 7:**
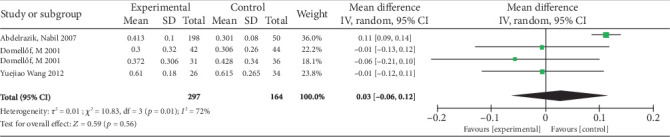
Forest plot for rate of weight gain.

Four articles were included in the analysis, demonstrating low statistical heterogeneity (*p* = 0.01, *I*^2^ = 72%). Meta-analysis using a random-effects model revealed no statistically significant effect of 1 mg/kg/day iron supplementation compared to placebo/no supplementation on weight gain (MD = 0.03, 95% CI −0.06 to 0.21, *p* = 0.56), and the forest plot is illustrated in Figure 7. Subgroup analysis indicated potential racial variations, with a marginal improvement trend observed in Asian and African populations (MD = 0.07 kg/month) (Table 2).

## 4. Discussion

Iron supplementation during 4–6 months of age significantly increased Hb levels (MD = 6.29 g/L, *p* = 0.01) Treatment efficacy may be influenced by the type of iron (e.g., preliminary data suggest iron dextran might be more effective than ferrous sulphate), geographic iron stores, and baseline iron status. The observed difference in efficacy between iron formulations requires cautious interpretation due to the limited number of comparative studies and small sample sizes, as noted in the limitations. Furthermore, given that the prevalence of ID and anemia is commonly higher in developing countries than in developed ones, nations are often categorized as having relative ID or sufficiency based on this epidemiological pattern [16]. In iron-deficient regions, iron supplementation resulted in a nonsignificant increase in Hb of 5.64 g/L (*p* = 0.12), possibly due to poor baseline iron status, nutritional deficits, infections, or small samples. In contrast, a study in an iron-sufficient setting reported a significant 8.20 g/L Hb rise (*p* < 0.001), likely attributable to better iron stores and targeted supplementation. These findings are limited by study heterogeneity and a single included study in iron-sufficient populations, necessitating further validation. The supplementary treatment demonstrated a statistically significant increase in SF levels at 12 months (MD = 5.95 *μ*g/L, 95% CI 0.23–11.58, *p* = 0.04), with no significant effect observed at 6 months. However, extended supplementation up to approximately 12 months of age yielded no sustained benefits for Hb (MD = 2.00 g/L, *p* = 0.08), with minimal impact on growth parameters (only slight elevation of weight gain in the Asian and African populations). This delay likely reflects the prioritization of iron utilization: in the short term (4–6 months), supplemented iron is primarily diverted to erythropoiesis to maintain Hb levels, with minimal surplus for storage. Over longer periods (up to 12 months), as Hb homeostasis is achieved, excess iron can be stored in ferritin, explaining the delayed rise in SF. However, the limited number of studies evaluating SF (only three included) and their small cohort sizes (e.g., the 6-month analysis relied on a single underpowered study) may have obscured earlier effects, as small samples have insufficient statistical power to detect modest but clinically relevant changes in storage iron. These findings suggest significant variation in iron supplementation efficacy based on intervention duration, iron formulation, baseline iron status, and regional deficiency prevalence. Hb improvement appears most pronounced in high-prevalence ID regions, while effects remain limited in areas with adequate iron stores. The small number of included studies (less than 10) precluded publication bias assessment and sensitivity analyses.

The current findings demonstrate different from a 2017 meta-analysis examining early iron supplementation in breastfed infants [17]. The cited study reported no significant effects of iron intervention on SF and Hb levels, though mean red blood cell volume showed marked elevation. While iron supplementation exhibited no impact on infant length, it significantly suppressed both weight gain and head circumference growth, potentially attributable to variations in iron dosage and intervention duration across included studies. Discrepancies between the outcomes of this study and prior reports suggesting iron supplementation reduces weight and head circumference gain likely arise from nonstandardized dosing regimens [18]. In contrast, our protocol strictly adheres to the majority of current guidelines by administering iron at a precisely controlled dose of 1 mg/kg/day. This discrepancy is likely attributable to our strict inclusion criteria focusing solely on the 1 mg/kg/day dosage, which minimized the confounding effects of heterogeneous dosing regimens present in earlier reviews. Evidence suggests comparable efficacy between daily and weekly iron supplementation for infant IDA prevention [19]. Early initiation at 3 months of age demonstrates significantly lower ID and IDA prevalence compared to exclusively breastfed infants receiving no intervention. An article included in this meta-analysis also mentioned [12] that neither daily nor weekly iron supplementation for 3 months in healthy 4-month-old exclusively breastfed infants prevented ID or IDA. This intervention also failed to alter overall Hb levels or iron stores. Consequently, the current analysis examines whether weekly supplementation could substitute daily iron supplementation to reduce gastrointestinal adverse effects and improve adherence. A 2024 JAMA publication [10] demonstrated that 1 mg/kg/day iron supplementation in exclusively breastfed infants from 4 to 9 months of age produced no psychomotor development benefits during the first 3 years of life. Iron homeostasis depends on the equilibrium between iron stores, intake, and consumption (loss) of the organism. ID develops when stores and intake become inadequate to compensate for increased losses or demand, failing to support normal growth and development [20]. During gestation, fetal iron acquisition occurs primarily through placental active transport, with approximately 60% from the last 3 months of the month. Total iron content of the organism at birth averages 75 mg/kg. Postnatal erythrocyte dissolution releases iron previously contained within Hb (3.47 mg/g). Delayed umbilical cord clamping (2–3 min) provides approximately 75 mg additional elemental iron from Hb, sufficient for approximately 3 months of iron requirements [21]. These neonatal iron reserves typically maintain adequate status for the first 6 postnatal months.

Based on this meta-analysis, the following recommendations were propounded: (1) High-risk populations (including maternal anemia, preterm birth, or low birth weight infants) should follow AAP recommendations and require iron supplementation at 2 mg/kg/day from 1 month after birth to 12 months of age if exclusively breastfed. If they are formula-fed infants, 1.8–2.2 mg/kg/day can be obtained from milk powder. Since 14% of preterm infants remain iron deficient at 4–8 months of age despite the use of iron-fortified formula, partially formula-fed preterm infants may require additional iron supplementation. (2) Low-risk groups: According to the ESPGHAN, exclusive breastfed or primarily breastfed healthy full-term infants are recommended for iron supplementation at a dose of 1 mg/kg/day initiated at 4 weeks of age (1 month) and discontinued when the infant can obtain sufficient iron through complementary foods (usually around 6 months of age). (3) In resource-sufficient areas, screening for Hb and SF should be recommended for iron supplementation at 6 months of age. This analysis has several limitations. First, there is heterogeneity in interventions, including variations in iron formulations and supplementation durations across studies. Second, there is insufficient long-term developmental data, with only two studies reporting outcomes up to 3 years of age. Third, there is a lack of stratification by baseline iron status in most included studies. Fourth, the total number of included studies is small, with some individual studies having relatively small sample sizes. For instance, the analysis of SF levels at 6 months depends solely on one underpowered study. Such insufficient sample sizes hinder the detection of subtle yet clinically meaningful effects. Subgroup analyses on iron formulations, ethnicity, and ID severity are based on very few studies (some subgroups include only 1–2 studies), reducing result stability and reliability due to inadequate statistical precision. Additionally, small sample sizes limit robust sensitivity analyses, as minor changes in included studies may significantly alter overall effect estimates.

## 5. Conclusion

This study represents the first systematic evaluation and meta-analysis comprehensively assessing the effects of 1 mg/kg/day iron supplementation on iron status indicators (Hb, MCV, SF) and growth parameters (height, weight) in exclusively breastfed full-term infants. The findings provide crucial evidence for optimizing individualized iron supplementation protocols, balancing the risks of IDA prevention and overintervention, and informing global guideline updates and clinical practice standardization. Short-term (4–6 months) 1 mg/kg/day iron supplementation significantly increased Hb levels but provided no sustained benefit with longer-term use. For SF, this dosage showed no increase at 6 months; a delayed elevation occurred at 12 months, though this requires validation in larger studies due to small sample sizes. In low anemia-risk settings, targeted monitoring and supplementation strategies demonstrate greater cost-effectiveness, whereas high-risk areas still require standardized protocols. Clinical decision-making must balance geographic epidemiologic characteristics, testing resources, and parental adherence to precision nutrition principles. Future large-scale, high-quality RCTs with longer follow-up periods are warranted to confirm the long-term developmental effects of iron supplementation and to identify reliable biomarkers for guiding individualized supplementation.

## Figures and Tables

**Table 1:** Characteristics of included studies.

**Number**	**Author**	**Year**	**Stata**	**Research design**	**Sample size (experimental/control group)**	**Experimental group intervention/control group intervention**	**Duration of intervention**	**Outcome measurement indicators**
1	Domellöf et al.	2001	Honduras, Sweden	Randomized controlled trial	156 (76/80)	(Ferrous sulfate) **e**lemental **i**ron 1 mg/kg/day/control: Placebo	Iron supplementation at 4–9 months of age	Hb, MCV, SF, ZPP, STfR, and body weight in April and September were measured at 4, 6, and 9 months of age
2	Yurdakok et al.	2004	Turkish	Randomized controlled trial	52 (27/25)	(Ferrous sulfate) iron 1 mg/kg/day/control group: No iron supplementation	Iron supplementation at 4–7 months of age	Hb, MCV, RDW, TS, and SF at 4, 5, 6, and 7 months of age
3	Abdelrazik et al.	2007	Egypt	Randomized controlled trial	248 (198/50)	(Ferrous gluconate) elemental iron 1 mg/kg/day/control: Placebo	Iron supplementation from 4 to 6 months	Weight, length and head circumference gained at 6 year and 12 months postintervention
4	Gokcay et al.	2012	Turkish	Randomized controlled trial	105 (51/54)	(Ferrous sulfate) iron 1 mg/kg/day/control group: No iron supplementation	Iron supplementation at 6–12 months of age	Hb, SF at 12 months of age
5	Wang et al.	2012	China	Randomized controlled trial	123 (63/60)	(Amino acid chelated iron) iron 1 mg/kg/day/control group: No iron supplementation	Iron supplementation from 4 to 6 months of age	Height and weight gain at 6 months of age
6	Wang et al.	2012	China	Randomized controlled trial	209 (107/102)	(Iron dextrose) elemental iron 1 mg/kg/day/control group: No iron supplementation	Iron supplementation from 4 to 6 months of age	Hb at 6 months of age
7	Svensson et al.	2024	Poland, Sweden	Randomized controlled trial	221 (111/110)	(Micronized microencapsulated ferric pyrophosphate) elemental iron 1 mg/kg/day/control: Placebo	Iron supplementation at 4–9 months of age	Hb, SF at 12 months of age

**Table 2:** Subgroup analysis.

**Indicators of outcome**	**Subgroup classification**	**Number of studies**	**Heterogeneity test**		**Effect model**	**Meta**	
			**p**	**I** ^2^		**MD (95% CI)**	**p**
4–6 months' intervention, Hb at 6 months of age	*Type of iron*						
	Ferrous sulfate	3	*p* = 0.005	81%	R	4.25 (−0.42, 8.92)	*p* = 0.07
	Dextran iron	1	—	—	—	12.71 (8.77, 16.65)	*p* < 0.001
	*Ethnicity*						
	Afro-Asian	2	*p* < 0.001	95%	R	6.34 (−6.12, 18.79)	*p* = 0.32
	Europe and America	2	*p* = 0.11	61%	R	6.33 (2.52, 10.15)	*p* = 0.001
	*Sample size*						
	Large samples (*n* > 50)	3	*p* = 0.008	79%	R	8.33 (3.84, 12.83)	*p* = 0.0003
	Small samples (*n* ≤ 50)	1	—	—	—	0.00 (−3.74, 3.74)	*p* > 0.999
	*Severity of iron deficiency*						
	Countries with relative iron deficiency	3	*p* < 0.001	91%	R	5.64 (−1.45, 12.73)	*p* = 0.12
	Relatively iron-sufficient countries	1	*—*	—	—	8.20 (5.03, 11.37)	*p* < 0.001

MCV	*Ethnicity*						
	Afro-Asian	1	—	—	—	2.50 (1.03, 3.97)	*p* = 0.0009
	Europe and America	2	*p* = 0.25	26%	F	0.56 (−0.70, 1.82)	*p* = 0.38

Rate of weight gain	*Ethnicity*						
	Afro-Asian	2	*p* = 0.05	75%	R	0.07 (−0.04, 0.18)	*p* = 0.24
	Europe and America	2	*p* = 0.62	0	F	−0.03 (−0.12, 0.07)	*p* = 0.61

## Data Availability

The dataset generated for this study is available upon request to the corresponding author.
